# Nanoparticle Skin Penetration: Depths and Routes Modeled In‐Silico

**DOI:** 10.1002/smll.202412541

**Published:** 2025-03-27

**Authors:** Natsumi Maeda, Haixin Jiao, Ilona Edyta Kłosowska‐Chomiczewska, Wojciech Artichowicz, Ulrich Preiss, Patrycja Szumała, Adam Macierzanka, Christian Jungnickel

**Affiliations:** ^1^ Institute of Biogeochemistry and Pollutant Dynamics Swiss Federal Institute of Technology ETH Zürich Universitätstrasse 16 Zürich 8092 Switzerland; ^2^ Biofuels Institute School of the Environment and Safety Engineering Jiangsu University Zhenjiang 212013 P. R. China; ^3^ Department of Biotechnology and Microbiology Faculty of Chemistry Gdańsk University of Technology Narutowicza 11/12 Gdańsk 80–233 Poland; ^4^ Department of Hydraulic Engineering Faculty of Civil and Environmental Engineering Gdańsk University of Technology Narutowicza 11/12 Gdańsk 80–233 Poland; ^5^ Sustainability Omya GmbH Siegburger Str. 229c 50679 Köln Germany

**Keywords:** nanoparticles, pharmaceutical delivery, QSPR, rational division, skin penetration

## Abstract

Nanoparticles (NPs) are increasingly explored for targeted skin penetration, particularly for pharmaceutical and cosmetic applications. However, the complex system between NP properties, skin structure, and experimental conditions poses significant challenges in predicting their penetration depth and pathways. To what depth do NPs penetrate the skin, and which pathways do they follow? These are the questions which we tried to answer in this paper. A n in‐silico human skin model based on 20 years of literature on NPs skin penetration is developed. The model incorporates 19 independent parameters, including a wide range of NP properties, skin across species, and test conditions. Using random forest analysis coupled with Kennard‐Stone sorting, the model achieves a high predictive accuracy of 95%. The study identifies hair follicle diameter as the most critical factor influencing NP penetration across skin layers, surpassing other skin properties, NP properties, or experimental variables. Pig and rabbit skin are the most suitable models for simulating human skin in NP penetration studies. Additionally, the in‐silico model reveals that NPs in emulsions and oil‐based media predominantly follow the intercellular and transappendageal route. In contrast, those embedded in aqueous media favor the intracellular route. These findings offer insights for optimizing NP‐based drug delivery systems.

## Graphical abstract

An in‐silico human skin model based on 20 years of NP penetration studies, incorporating 19 parameters is developed. Using random forest and Kennard‐Stone sorting, the model achieves 95% accuracy, identifying hair follicle diameter as the dominant penetration factor. NP penetration pathways depend on the medium, with emulsions favoring intercellular/transappendageal routes and aqueous media favoring intracellular penetration.

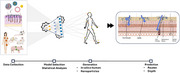

## 1 Introduction

Extensive research on engineered nanomaterials has been conducted better to understand their behavior, properties, and potential applications. These nanomaterials are ubiquitous in everyday products such as cosmetics, pigments, pharmaceuticals, textiles, food packaging, agriculture, water treatment, electronics, and catalysts. It includes organic nanomaterials like fullerenes, carbon nanotubes, graphene, polymers, micelles, and dendrimers. But also metal‐based nanomaterials such as silver (Ag) and copper (Cu), metal oxides like zinc oxide (ZnO) and titanium dioxide (TiO₂), and other types of materials such as quantum dots and titanium carbide. NPs, a subset of nanomaterials, exist in various shapes, including spherical, amorphous, and crystalline forms.^[^ ^1^ ^]^ NPs are generally classified into two categories: naturally occurring (e.g., volcanic ash, soil colloids, proteins, viruses, and antibodies) and engineered. The engineered NPs are specifically created for industrial and technological applications, which forces further research as regulatory pressures increase with growing awareness.^[^ ^2^ ^]^ In this study, we use the term NP to refer to engineered NPs. The NPs included in this study are further discussed in the Experimental Section.

NPs' unique properties stem from their small size, which results in a high surface‐to‐volume ratio and a change in the chemical potential difference between particles and surrounding media. Researchers have leveraged these properties in transdermal delivery to explore how NPs can penetrate different skin layers tailored to specific therapeutic or cosmetic purposes. Over the past two decades, increasing numbers of experimental studies have examined the interactions between NPs and the skin cellular structures (Figure S1, Supporting Information) and their effectiveness in transdermally delivering pharmaceuticals and other bioactive molecules.

The penetration depth is a key factor in applying NPs. Sunscreens containing NPs likeTiO_2_ and zinc oxide ZnO are designed to remain on the skin surface. They scatter and reflect UV rays,^[^ ^3^ ^]^ avoiding the absorption of dissolved inorganic metals and their adverse effects.^[^ ^4^ ^]^ In contrast, drug‐delivery NPs are engineered to penetrate specific skin layers. For instance, topical hydrocortisone should target the dermis to treat dermatitis effectively, but excessive penetration into the bloodstream can lead to systemic side effects, including hypothalamic‐pituitary‐adrenal suppression and iatrogenic Cushing's syndrome.^[^ ^5^, ^6^ ^]^ Similarly, silver sulfadiazine is applied to burn wounds with the aim of local antimicrobial action, but deeper absorption risks silver deposition and organ damage.^[^ ^7^ ^]^ Precise epidermal penetration is critical in other cases, as with idoxuridine, which treats herpes simplex keratitis by reaching infected layers.^[^ ^8^ ^]^

Transdermal delivery of pharmaceuticals and other bioactive molecules offers several advantages over conventional routes such as intravenous, intramuscular, or transmucosal pathways. Bypassing hepatic first‐pass metabolism provides a non‐invasive alternative that enhances patient compliance through sustained drug release, reducing the dosing frequency, minimizing toxicity risks, and lowering overall treatment costs. Furthermore, it improves bioavailability as drugs enter the bloodstream in active forms and offers an alternative option for patients unable to take medications orally due to nausea or gastrointestinal distress.^[^ ^9^, ^10^, ^11^ ^]^

**Figure 1:**
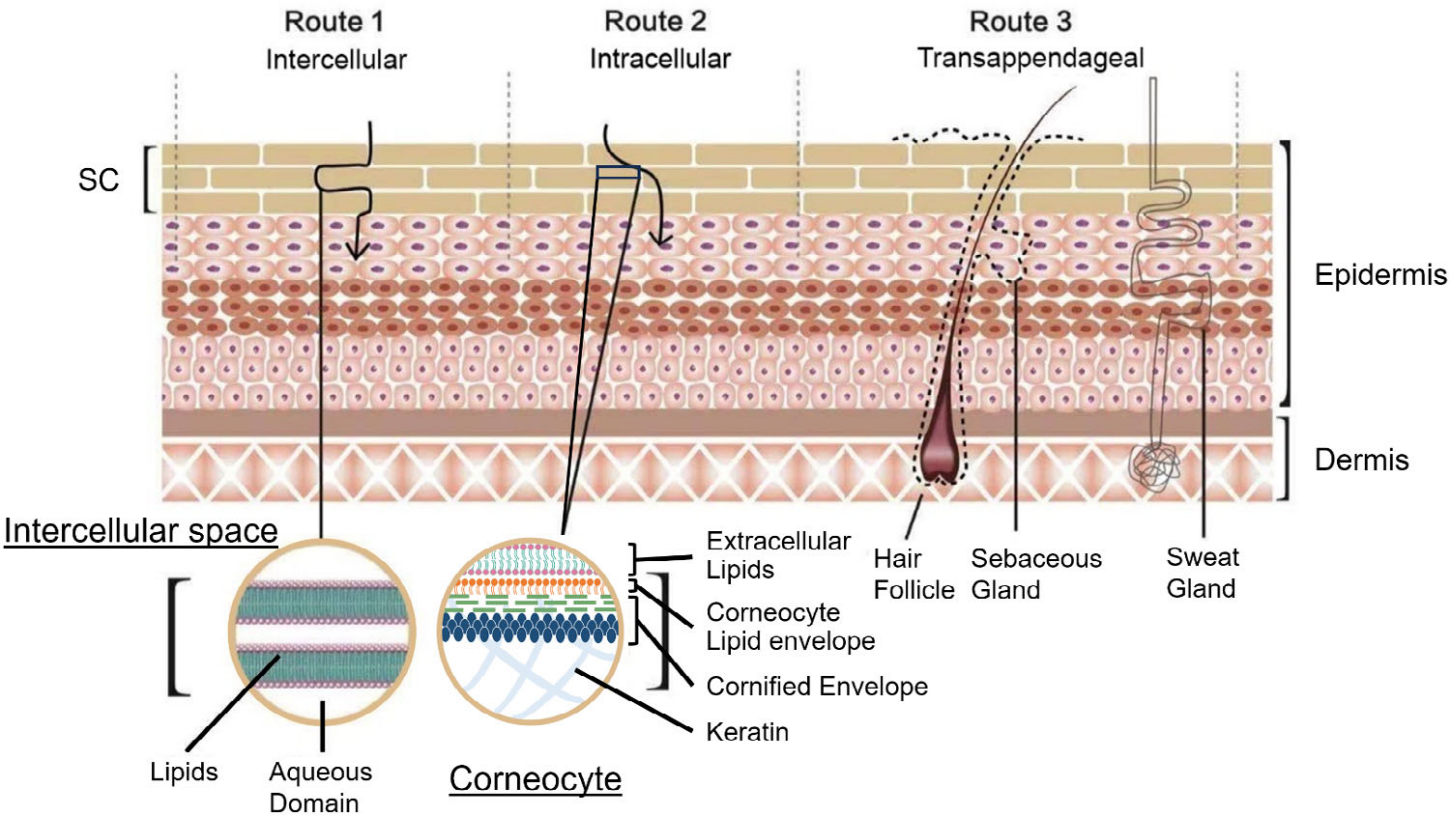
Schematic representation of the skin. The *stratum corneum* consists of corneocytes (dead keratinized cells), comprising a corneocyte lipid envelope and a cornified envelope composed of various proteins and internal keratin. The intercellular matrix consists of a lamellar, liquid‐crystal structure with an aqueous domain surrounded by lipids. It illustrates the three penetration routes of NPs: A) intercellular, B) intracellular, and C) transappendageal. Elements are drawn with the assistance of BioRender.

However, the ability of NPs to bring therapeutic compounds to a target location using transdermal routes is often limited by the skin's outer barrier cornified layer, that is, *stratum corneum* (SC).^[^ ^12^, ^13^ ^]^ This layer exhibits a “brick‐and‐mortar” architecture, where corneocytes (flattened, dead keratinocytes) serve as the “bricks”, while the intercellular lipid matrix acts as the “mortar”, as illustrated in **Figure** **1**. Corneocytes are flattened, dead keratinocytes composed of a monolayer corneocyte lipid envelope and a cornified envelope made of various proteins (e.g., involucrin and loricrin), along with internal keratin.^[^ ^14^, ^15^ ^]^ The intercellular cement is a lamellar, liquid‐crystal‐like structure made primarily of ceramides, cholesterol, free fatty acids, other lipids, and aqueous domains encased in the lipids.^[^ ^12^, ^16^, ^17^ ^]^ The anisotropic nature of this structure adds complexity to penetrate NPs through the skin.

Upon application of NPs to the skin, three main routes for further skin penetration are possible: intercellular, intracellular, and transappendageal. The intercellular pathway describes NPs diffusing through the lipid matrix surrounding the corneocytes in the SC, partitioning between the lipid bilayers and aqueous domain as they diffuse toward the deeper layers of the skin. The intracellular route passes directly through corneocytes in the SC and keratinocytes beneath the SC. Last, the transappendageal route facilitates the passage of molecules through hair follicles. These pathways are summarized in Figure 1.

Several methods have been proposed to reduce the barrier properties of the SC, classified as either active/physical or passive/chemical approaches.^[^ ^18^ ^]^ Active methods involve physically disrupting the SC or using external energy to enhance skin permeability. Techniques such as microneedles and jet injectors, along with ultrasound, thermal, and electrical‐assisted methods, facilitate drug transport through the skin.^[^ ^9^, ^19^, ^20^, ^21^ ^]^ In contrast, passive methods focus on modifying the SC structure through the use of chemical penetration enhancers such as alcohols, sulfoxides, and urea. These enhancers improve transdermal drug delivery by decreasing the viscosity of the lipid bilayers in SC, extracting intercellular lipids, increasing the hydration of the skin, or altering the structural proteins.^[^ ^22^, ^23^ ^]^ Another passive strategy involves formulations with drug‐loaded nanoscale carriers, like liposomes or micro‐ and nano‐emulsions, that penetrate the skin after being applied topically.^[^ ^18^, ^24^ ^]^

The common part of all relevant research is projecting experimental or literature data onto human skin as the primary model. Yet, the studies on the penetration of NPs employ a wide range of skin origins, including different species (i.e., humans, pigs, mice, rats, and rabbits), and different body regions. The literature data implies that penetration depth varies due to differences in skin layer thickness, density of hair follicles, and lipid composition, making direct comparisons difficult.^[^ ^25^ ^]^

Additionally, experimental systems vary in parameters such as temperature, in vivo versus ex vivo condition, contact time of NPs to skins, the use of perfusate, and detection methods, which add further complexity to the standardization and comparison.

Given the intricate system involving different types of NPs, the skin of varying origin, region, and structure, as well as the diversity of experimental conditions, predicting NPs' skin penetration depths and routes is challenging. A couple of research groups have conducted in‐silico analyses of NP transdermal delivery, focusing specifically on the interaction between NPs and lipid membranes or the influence of NP properties such as size, shape, and surface chemistry.^[^ ^26^, ^27^, ^28^, ^29^ ^]^ In contrast, our study sought a more holistic perspective on NP skin penetration. We established three primary objectives in this study. The first is to propose an inter‐species model that can analyze NP skin penetration with the inclusion of species‐specific details. The second objective is to identify the key parameters influencing NP skin penetration. The final goal is to develop a predictive model for both skin penetration depth and routes of NP. To realize these objectives, we have compiled extensive data from the scientific studies on NP skin penetration in various species conducted in the past 20 years. Moreover, additional data on skin properties from these species and body regions were gathered, expanding our dataset for skin modeling and finally enabled to project the model onto human skin regions, essentially allowing us to create an in‐silico human skin model. This model allowed us to perform a broad range of NP skin penetration tests in our study.

To our knowledge, a comprehensive in‐silico study that considers all these factors (NP characteristics, skin type variations, skin properties, and a range of experimental conditions) has not yet been conducted while predicting the penetration depth and pathways. Therefore, this study introduces the first predictive model based on experimental data that thoroughly investigates NP transport through the skin.

## 2 Experimental Section

### 2.1 Data Collection

**Figure 2:**
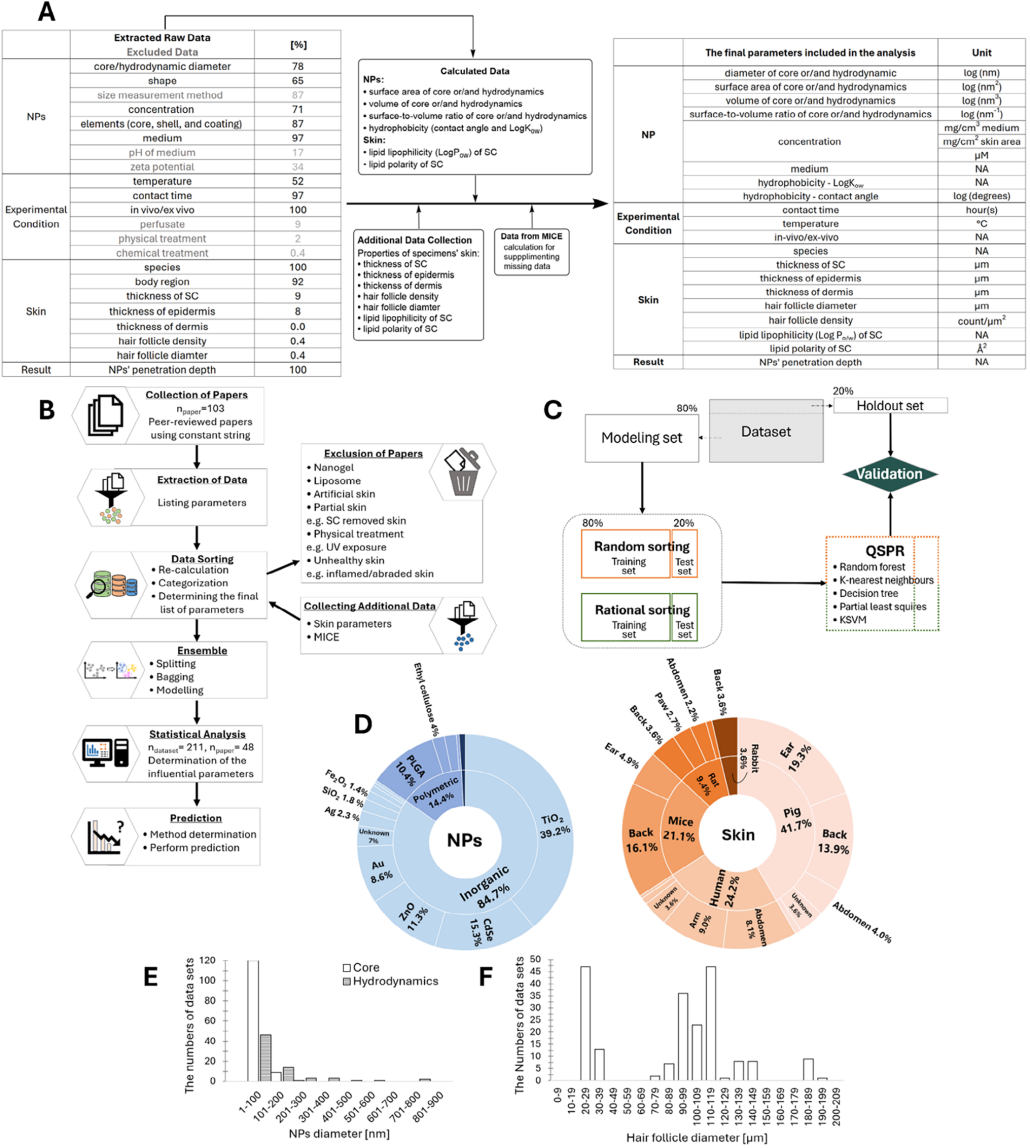
The summary of methodologies and parameters is compiled in the model. A)Parameters were extracted from published, peer‐reviewed scientific articles reporting NP skin penetration data (left table). The percentages indicate how many papers included each corresponding parameter out of the total. Some parameters were excluded from the analysis (in gray) due to insufficient data or merged in other parameters; details in the Experimental Section. Additionally, some NP and skin data were further calculated to generate new variables, such as surface area, derived from the diameter and shape of the NPs used in the studies. To fill the missing data, Supporting Information was gathered from additional, newly collected papers. The final dataset used in our model is presented in the table on the right. The log of NP parameters was used to maintain a comparable order of magnitude between variables for the models, for more robust models. More details were discussed elsewhere in the Experimental Section. B) Flowchart illustrating the workflow of our study. C) Schematic representation of the dataset splitting for modeling. D) Distribution of NPs and skin types in the collected studies. E,F) The distribution of NP core/hydrodynamic diameters and hair follicle diameter, respectively, included in our datasets. MICE: Multivariate imputation by chained equations. QSPR: quantitative structure–property relationship. ksvm: k‐class support vector machines.

Data was collected by first gathering published peer‐reviewed scientific articles on Google Scholar.^[^ ^30^ ^]^ To select papers of interest, a consistent search string *“skin penetration” AND “NP” AND “stratum corneum” AND “histology” ‐microneedle ‐reviews ‐NLC ‐SLN ‐liposome ‐DNA ‐RNA “vivo” OR “vitro”* was used to search each calendar starting in 2023, and going backward until no more papers were found. Using a consistent search string helps avoid the risk of missing correlated research, as opposed to relying on multiple individual keywords. This search string, using Boolean operators (AND, OR, and ‐), ensured that the results included comprehensive studies but focused on NP skin penetration while filtering out irrelevant topics such as microneedles, review articles, and specific NP types (nanostructured lipid carriers (NLC), solid lipid nanoparticles (SLN), liposomes, DNA, and RNA‐based NPs).^[^ ^31^, ^32^ ^]^ Experiments that analyzed skin penetration with liposome, nano gel, NLC, and SLN were excluded from the analysis as the incompatibility with other NP due to its size changes over time.^[^ ^33^ ^]^ Experimental data with artificial or model skin and pathological skin (e.g., UV irradiated skin, skin with cancer, allergies, inflammation, abrasion) were also excluded. Only healthy, intact skin was considered, and without the prior removal of skin layers. The test data using specimens with exfoliated hair or genetically hairless specimens such as nude mice were also excluded from our data set. This filtering process ensured that the analysis focused exclusively on healthy skin with hair, including cut or shaved specimens, to avoid discrepancies in NP penetration under varying conditions. The types of NP and skin used in this study are summarized in **Figure** **2**D.

A total of 103 experimental studies reporting measured skin penetration depth of NPs were collected. From these papers, data for 21 descriptors (independent parameters) and NP penetration depths (dependent parameters) were extracted. One of the aims of this study was to identify common patterns in the reported data, which was highly scattered due to varying testing conditions, skin types, and NPs and their interactions. The selected parameters were intended to capture these variations and their influence on NPs' skin penetration depth. After thorough screening, re‐calculation, and data augmentation, a set of 19 independent variables (eight related to NP, three to the experimental conditions, and eight to skin properties) was finalized to represent the different parameters affecting penetration depth. The initial parameters provided by authors of literature publications, the final parameters considered for the modeling, and supplemental data are listed in Figure 2A. The workflow of the data collection, extraction, classification, and analysis is depicted in Figure 2B. Some of the missing data were estimated using the multivariate imputation by chained equations (MICE), described in detail in Ref. [34]. Each missing value was briefly predicted using a regression model based on available data. This process was repeated multiple times to refine the estimates. It was found that eight imputations minimized the error and were thus applied.

#### 2.1.1 Parameters Describing NPs

The initial NP parameters selected for analysis included diameter, surface area, volume, surface‐to‐volume ratio, shape, concentration, logK_ow_, contact angle, elemental composition (core, shell, and coating), medium, pH, zeta potential, and the methods used for size measurement. Surface area, volume, and surface‐to‐volume ratio were calculated based on NPs' diameter and shapes. If NP shape was not provided in the original publication, and after verifying available product descriptions, a spherical shape was assumed, as this was the most common. In this study, the surface‐to‐volume ratio reflected the size of the NPs and shapes. As NP size decreased, the material properties changed significantly, resulting in a larger reactive surface area; for example, non‐magnetic metals could become magnetic, and noble metals might exhibit catalytic properties at the nanoscale.^[^ ^35^ ^]^ The size measurement method was noted to clarify whether the reported NP size referred to the core or the hydrodynamic diameter. Figure 2E shows the size distribution of NPs collected for modeling, with the majority falling within the ISO‐defined range of up to 100 nm.^[^ ^36^ ^]^

Many of the media, such as aqueous (such as simulated sweat, or buffer) or oil, were homogeneous. However, some of the NPs media were heterogeneous, consisting of mixtures like sunscreen formulations or commercially available cosmetic emulsions. Unfortunately, other independent variables describing the medium, such as pH and zeta potential, were insufficiently reported in the papers—only 17% and 34%, respectively. As a result, imputing these values using MICE was not possible, so they were excluded from this dataset and further analysis. Instead, the media was categorized by broader qualitative classifications such as aqueous, oil, or emulsion (oil in water), as the majority fell into those categories. This approach allowed the authors to capture some variations among those groups and assess their potential effects on the penetration. The NPs were categorized as inorganic, polymeric, or other based on their description and core material. As a final parameter, only the outermost layer's hydrophobicity, measured as logK_ow_ for organic NPs or contact angle for inorganic NPs, was included since it is primarily responsible for interactions with the surrounding environment. The authors used the Molinspiration online calculator for organic compounds, as its consistent algorithm for calculating logK_ow_ was accepted, which provided the most reliable standardization across compounds. Unfortunately, a similar approach was not feasible for inorganic compounds; thus, if not explicitly provided in the source papers, the contact angles were gathered separately. In the end, eight parameters describing NPs were selected for further analysis: diameter, surface area, volume, surface‐to‐volume ratio, concentration, medium, and hydrophobicity (measured as either logK_ow_ or contact angle).

#### 2.1.2 Parameters Describing Experimental Conditions

Initially, the following parameters describing experimental conditions were noted: testing system temperature, contact time of NP with the skin, in vivo versus ex vivo conditions, perfusate presence, and any physical or chemical treatments applied. Ultimately, only three parameters were included in the final analysis, that is, temperature, contact time, and if the experiment utilized in vivo/ex vivo skin. Perfusate was excluded because these solutions did not contain NPs and primarily served to mimic bodily fluids. While they could slightly alter NP concentration during skin penetration studies, their primary role was to simulate physiological conditions, not to interact directly with the NPs. Additionally, studies involving physical or chemical pre‐treatments were excluded due to the limited number of consistent data reported in such experiments (Figure 2A).

#### 2.1.3 Parameters Describing Skin

The collected papers on NP skin penetration tests involved a variety of specimens, including humans, pigs, mice, rats, and rabbits (Figure 2D Skin). To account for differences in skin types, several skin‐related parameters were incorporated into this model: the species used (e.g., human, pig, rabbit), regions of skin (e.g., arm, back, breast), the thickness of the SC, epidermis, and dermis, hair follicle density and diameter, as well as the lipophilicity and polarity of lipids in the SC. Since the experimental data on these skin characteristics was often not reported in the collected studies (Figure 2A), almost all skin‐related information was separately gathered based on the external, species‐ and body region‐specific data. For this purpose, an additional 65 papers, resulting in 135 datasets, were gathered from the scientific literature (Table S2, Supporting Information). At least three different data points were collected and averaged for each body region of the test specimens to assign relevant skin data to the NP penetration tests. Data on skin lipophilicity and polarity were obtained from nine studies specific to each species by collecting information on SC lipids' types (e.g., cholesteryl sulfate, free fatty acids, triglycerides) and mass percentages (Table S2, Supporting Information). Lipophilicity and polarity were quantified using SwissADME^[^ ^37^ ^]^ based on SMILES strings for the identified lipids. Each lipid's lipophilicity and polarity values were then calculated according to the weighed mass percentage in the specimen's SC. Although polarity and lipophilicity were often inversely correlated, they were not synonymous. Thus, both parameters were included to enhance the precision of our predictions.

The depth of NP skin penetration was categorized into five theoretical layers, including cases of no penetration and penetration beneath the skin, as follows: A) Surface: no penetration, B) SC, C) Epidermis, D) Dermis, and E) Distant: beneath the dermis, potentially into the bloodstream. The layer where most NPs accumulated was recorded as penetration depth. Although SC was technically part of the epidermis, it was classified as a separate layer due to its distinct physicochemical properties^[^ ^38^ ^]^ (Figure 1). It was important to note that penetration into hair follicles was classified as dermis penetration. This was because determining the specific route of dermal penetration, whether through hair follicles or via intracellular/intercellular pathways, was difficult, given that hair follicles extended from the skin surface deep into the dermis. The distribution of hair follicle diameters that were included in this study is depicted in Figure 2F.

### 2.2 Data Splitting

The collected data were randomly split into modeling and hold‐out (external testing) sets (80%:20%). The modeling set (80%) was further divided into training and validation sets (ratio, how many) using random and various rational division methods. The hold‐out validation splitting solved the over‐fitting problem common in resubstitution validation methods.^[^ ^39^ ^]^ Resubstitution validation was a commonly used technique where all the data were used for training, and the evaluation was calculated by comparing the outcome with an observed value.^[^ ^40^ ^]^ The hold‐out set played the role of observed data to compare with it since it was never used for training the model (Figure 2C). The random division was the commonly used method to split data;^[^ ^41^ ^]^ however, it was found that random division failed when datasets were small; however, it was found that it fails when datasets were small;^[^ ^34^ ^]^ there was always a risk that some descriptors would not be present in the training set and thereby decreased the accuracy of the model. An alternative method was a rational division, which ensured the inclusion of all descriptors and resulted in a higher applicability domain.^[^ ^42^ ^]^ Here, three rational division techniques were applied, namely, Kohonen self‐organizing maps (SOM), k‐means clustering (k‐means), and the Kennard‐Stone algorithm (KS). Those three methods were the most efficient solutions for small datasets.^[^ ^43^, ^44^, ^45^ ^]^ Kohonen SOM was an artificial neural network that clustered input patterns into groups,^[^ ^45^ ^]^ where the high‐dimensional input of the dataset would be transformed into a 2D output, maintaining the same relationship among the data points.^[^ ^46^ ^]^ K‐means clustering was based on partitioning a set of points into clusters, represented by an adaptively changing centroid. K‐means computed the least squared Euclidian distances between the points and centroids and assigned data to the nearest centroid.^[^ ^43^, ^47^ ^]^ The Kennard‐Stone algorithm was a technique that considered all points as candidates for the training set and chose them sequentially, starting from the highest Euclidean distance between two points.^[^ ^45^, ^48^ ^]^ Kohonen's self‐organizing map was carried out in the R implementation within XLSTAT, where the alpha was set between 0.01 and 0.05 and the topology was hexagonal.^[^ ^49^ ^]^ K‐means clustering was set with classes ranging from 2 to 20. The final optimal number of clusters was determined the elbow method and was briefly explained by.^[^ ^50^ ^]^ The Kennard‐Stone algorithm used the prospectr package in R.^[^ ^51^, ^52^ ^]^

### 2.3 Computational Analysis

Quantitative structure–property relationship models (QSPR) were classification models used in chemical science, which served as powerful tools for predicting biological activity, physicochemical property, and toxicological responses of chemical compounds without any tests, using only computed properties, thereby enabling targeted in‐silico design of novel materials suitable for a wide range of applications.^[^ ^53^ ^]^ In this paper, five QSPR modeling tools were used: decision tree, random forest, *k*‐nearest neighbors (*k*‐NN), partial least squares (PLS), and *k*‐class support vector machines (KSVM). These five QSPR modeling tools were selected to ensure a balance between interpretability (decision tree, PLS), predictive power (random forest, KSVM), and versatility in handling nonlinear relationships and diverse data structures (k‐NN, KSVM). A decision tree was defined as a classification procedure that used a tree‐like model, which partitioned a dataset into smaller subdivisions based on a set of questions defined at each branch, where all the questions followed either another question or a final classification.^[^ ^54^, ^55^ ^]^ The decision tree had several advantages: it could handle multiple mechanisms of action, handle high‐dimensional data well, and ignore irrelevant descriptors. Random forest consisted of a combination of decision tree predictors where each predictor was uncorrelated. The class with the most votes became the final prediction.^[^ ^54^ ^]^ The *k*‐nearest neighbor was a non‐parametric algorithm based on the similarity of features in a dataset. Every point was classified by a majority vote of its neighbors.^[^ ^56^ ^]^ PLS was a method for modeling relations between sets of descriptors by creating an inferential model. It comprised regression and classification tasks, dimension reduction techniques, and modeling tools.^[^ ^48^, ^57^ ^]^ In PLS, the variable importance in projection could be determined, and the formula was shown in Equation (1) according to ref. [58].

(1) $$VIP_{j}=\sqrt{\frac{\sum_{f=1}^{F}w_{jf}^{2}\times SSY_{f}\times J}{SSY_{total}\times F}}$$

where in this research, the classification problem consisted of using a QSPR model for predicting the class (denoting a depth of skin penetration y) based on the set of explanatory variables (skin and NPs properties *x*); therefore w*_jf_* is the weighted value for *j* variable and *f* component, *SSY_f_* is the sum of squares of explained variance for the *f*_th_ component, and *J* is the number of x variables. *SSY_tota_*_l_ is the sum of squares of the total explained by the dependent variable, and *F* is the total number of components.

The support vector machine (SVM) classifier relied on constructing the possibly widest margin separating the different classes.^[^ ^59^ ^]^ This could be easily achieved in a linearly separable case. However, when considering the non‐linear relationship, the data points were projected into the new feature space. This required the application of kernel functions (and thus became KSVM). As a result, non‐linear relationships might unfold in the new feature space and become linear ones or close to such; thus, they became easily separable. In addition to the prediction accuracy, Cohen's kappa was also applied to evaluate these results. Cohen's kappa was a robust statistical method for comparing the reliability of various publications conducted by different research groups. The value of kappa, ranging from −1 to 1, showed the level of agreement of the data, where values ≤0 mean no agreement, and 1 is a perfect agreement.^[^ ^60^, ^61^ ^]^ The formula of kappa (*k*) calculated from the confusion matrix is shown in Equation (2).

(2) $$k=\frac{N\sum_{i=1}^{n}m_{i,i}\;-\sum_{i=1}^{n}\left(G_{i}C_{i}\right)}{N^{2}-\sum_{i=1}^{n}\left(G_{i}C_{i}\right)}$$

where *i* is the class number, *N* is the total number of classified values compared to the correct classification, *m_i_*, *i* is the number of values belonging to the correct class *i* that was also classified as class *i*, *C_i_* is the total number of predicted values belonging to class *i*, *G_i_* is the total number of correct classification belonging to class *i*.

The prediction results for each model were presented as a confusion matrix and given in the supporting information (Table S3, Supporting Information).

Due to the high dimensionality of the NP dataset, the applicability domain was determined using the convex hull approach.^[^ ^62^ ^]^ The convex hull was the smallest convex set that contained all the data points in any number of dimensions.^[^ ^63^ ^]^ In this work, the convex hull was determined using the Quickhull package in R,^[^ ^64^ ^]^ after the dimensionality was reduced using PCA. The data was visualized using the rgl package also in R.^[^ ^65^ ^]^ The results of the applicability domain are shown in the Supporting Information (Figure S5, Supporting Information). R scripts were produced with the assistance of ChatGPT.

After the best rational splitting and QSPR methodologies were determined, a table of 100 000 random variations between the minimum and maximum values of the NP descriptors was generated (essentially, a table of 100 000 random NP). Using the skin values for humans, the penetrated layer of each randomly generated NP was determined using the best rational splitting and QSPR methodology. The descriptors that were statistically differentiated at each layer were determined by the Kruskal–Wallis test with a *post hoc* Dunn pairwise comparison. This allowed the authors to determine the range of values for each descriptor that would enable that NP to penetrate a specific layer. An overview of the workflow is shown in Figure 2B.

## 3 Results and Discussion

### 3.1 Model

The collected data were first split into a hold‐out and modeling set, and further, the modeling set was split into a test and training set (presented in the Supporting Information), all using three rational division techniques, that is, Kohonen SOM, k‐means clustering (k‐means), and the Kennard‐Stone algorithm (KS). Next, the data were modeled using four modeling techniques, that is, PLS, decision tree, SVM, and Random forest. The summary of the results of the predictive quality of the four sorting methods and the four modeling techniques at different combinations is shown in **Table** **1**. The Random Forest algorithm demonstrated the highest predictivity, particularly when combined with the Kennard‐Stone data splitting method. Random Forest provides robustness against overfitting and effectively handles non‐linear relationships and noisy data.^[^ ^66^ ^]^ The hyperparameter optimization results for random forest are shown in Figure S2 (Supporting Information). The underperformance of alternative data‐splitting methods is due to their inherent limitations. K‐means clustering can overemphasize central cluster regions while neglecting edge cases or rare patterns.^[^ ^67^ ^]^ Similarly, Kohonen SOM reduces the data into a lower‐dimensional topological map, potentially losing subtle but essential distinctions between data points.^[^ ^68^ ^]^

**Table 1:** The results of the sorting techniques (listed in the columns) and numerical methods (listed in the rows) are presented as a percent accuracy to predict the hold‐out data. Kennard Stone and Kohonnen SOM performed best for the sorting methods, while Random Forest outperformed the other QSPR techniques.

[%]	Random	Kenndard stone	k‐means	Kohonnen SOM	Average
PLS	38	50	35	45	42
Decision tree	83	90	88	90	88
SVM	65	73	68	70	69
Random forest (500 trees)	92	95	95	95	94
Average	69	77	71	75	

### 3.2 Which Factors Influence NP Penetration?

The Kennard‐Stone sorting and Random Forest numerical methods were chosen for further analysis, as they provided the best accuracy to predict hold‐out data (Table 1). The model provided by this combination of techniques was analyzed toward the importance of the factors affecting penetration. However, it is essential to note that specifying parameters affecting NP skin penetration in isolation is unfeasible because factors are numerous and interdependent. The interdependence of the parameters is shown in the PCA analysis in Figure S3 (Supporting Information). These factors interact dynamically, making it challenging to separate their individual effects. Therefore, the comprehensive approach presented here is necessary, acknowledging the interdependent nature of these factors and prioritizing them based on their relative influence in optimizing NP engineering and design.

**Figure 3:**
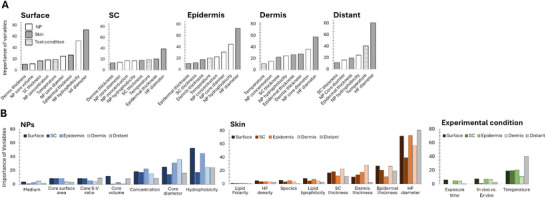
Importance of variable of NP skin penetration. A) Analysis including all parameters: properties of NPs, skin, and test condition, to reach the skin layers (Importance of variables > 10 is depicted). Hair follicle diameter was the dominant factor influencing NP skin penetration across all layers, followed by NP properties like hydrophobicity and core diameter, and skin thickness. Among test conditions, only temperature significantly affected penetration. The colors suggest parameters associated with the properties of NPs, Skin, and test conditions. HF: Hair Follicles. B) All parameters are categorized under NPs, skin properties, and experimental conditions. S–V: Surface‐to‐volume. Lipid hydrophobicity and Lipid polarity are lipids in SC.

The results of the analysis of NP skin penetration, highlighting the importance of all variables included in this study for each skin layer, are shown in **Figure** **3**A (only variables with importance greater than 10 are depicted). Hair follicle diameter emerged as the primary determinant of NP penetration across skin layers. This was followed by NP properties such as hydrophobicity and core diameter, and skin thickness. Among the experimental conditions, temperature was the only parameter with a significant effect on penetration. Figure 3B displays the analysis of NP skin penetration, showing the significance of all variables categorized under NP's properties, skin properties, and experimental conditions.

#### 3.2.1 Aggregation of NPs

Another challenge to efficient NPs skin penetration is aggregation. The aggregation process is commonly described by the Derjaguin–Landau–Verwey–Overbeek (DLVO) theory^[^ ^69^ ^]^ and other non‐DLVO forces (e.g., hydration and hydrophobic effects). Since we only observe NP penetration depth, the collected studies do not confirm whether aggregation occurred. Therefore, we cannot state the significance of aggregation.

In the DLVO theory, the interaction energy is determined by adding attractive van der Waals force (*V*_vdW_) and repulsion electrostatic double‐layer force (*V*_EDL_). According to equations presented by Elimelech et al.,^[^ ^70^ ^]^ the most significant parameters of *V*_vdW_ relevant to our study include the diameter of NP, properties of NP outer layer, medium, and temperature. For *V*_EDL_, key factors are the distance between particles and the Debye–Hückel parameter, which depends on the properties of the medium and temperature.

Aggregation occurs only when particles are close enough, making NP concentration and Brownian motion essential in facilitating their proximity. However, we cannot directly determine aggregation from the study results. Instead, its presence is inferred in later sections.

#### 3.2.2 NP Properties Effect

Parameters of NPs affecting transdermal transport are depicted in Figure 3B.

Hydrophobicity plays a key role in the penetration of NPs through skin layers. The surface hydrophobicity of NPs determines whether they remain on the skin surface or penetrate deeper into the epidermis by influencing both NP–NP interactions and NP–skin interactions. Hydrophobicity is a critical factor in NP aggregation; water disrupts the hydrogen bonding network in aqueous environments, increasing interfacial energy at the NP–water interface. To minimize this energy, NPs aggregate to reduce their exposed surface area to water.^[^ ^71^ ^]^ Attractive *V*_vdW_ drives aggregation even at larger distances.^[^ ^70^, ^72^ ^]^ Aggregated NPs remain on the surface due to size constraints. However, unaggregated hydrophobic NPs can passage through the intercellular route facilitated by SC lipids, aiding their penetration into the epidermis.^[^ ^73^ ^]^ Surface modification of NP, particularly with silica, is a technique gaining increasing attention,^[^ ^74^ ^]^ which enables even larger NPs (360 nm) to penetrate more effectively.^[^ ^75^ ^]^

**Figure 4:**
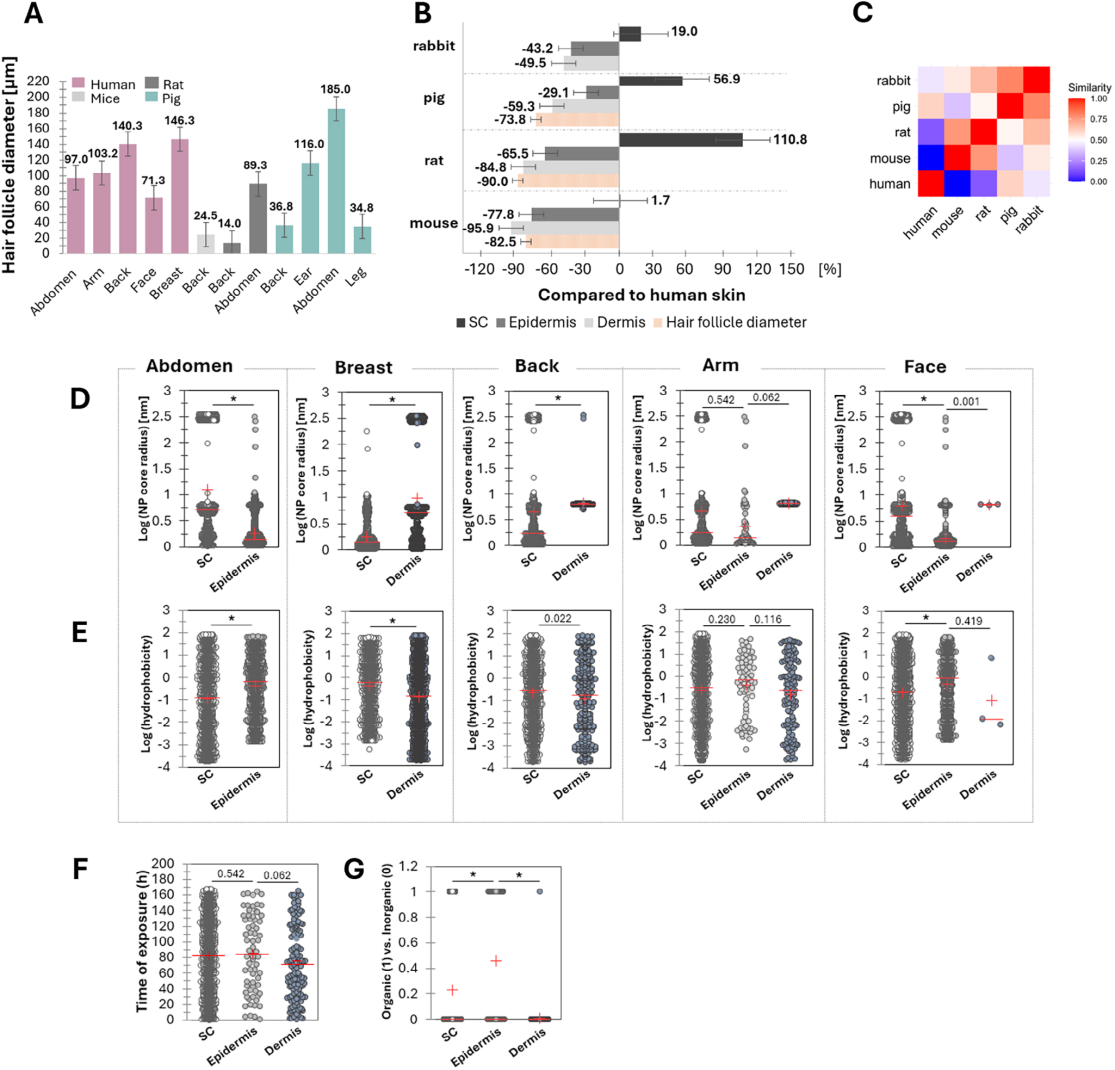
Skin differences across the species (A–C) and NPs penetration size‐ and hydrophobicity‐ dependence across various body regions (D–G). A) Hair follicle diameters of body regions across different species (human, mice, rat, and pig) included in the collected studies. B) Comparison of human skin depth (SC, epidermis, dermis) and hair follicle diameter of the back region with those of rabbit, pig, rat, and mouse. The numbers above the error bars depict the mean values. The data of hair follicle diameter of rabbit was not found thus, not depicted in this figure. C) The heatmap illustrates the similarity of skin thickness across different species in the back region. To assess the dermal thickness similarity, the Euclidian distance between values was determined by dist function in R. Pig skin is the closest to human skin, followed closely by a rabbit. D,E) Size‐ and hydrophobicity‐ dependent penetration depth of NPs in different skin areas, respectively. F,G) The impact of exposure time and the comparison of organic versus inorganic NPs on NP concentration in the skin of the arm, respectively. D–F) resulted using in‐silico generated 100 000 NPs and only the layers penetrated by NPs are shown. *p*‐values from the Kruskal–Wallis test for the parameters across different skin regions. * denotes *p*‐values < 0.0001.

The core diameter of NPs is a key factor in skin penetration, particularly for the delivery into the epidermis and dermis (Figure 3B, NPs). Diffusion routes can be considered anatomically: hair follicles, which extend into the dermis, have openings ranging from 14 to 185 µm depending on the species and body site (**Figure** **4**A). The intercellular route, situated between corneocytes filled with lipids, is ≈75 nm wide.^[^ ^76^ ^]^ Thus, only small NPs can traverse the intercellular spaces.^[^ ^77^ ^]^ These dimensions represent the theoretical upper limits for diffusion through these pathways. However, it should be noted that our study included both hard and soft NPs, therefore, size buffers must be considered in the case of soft NPs capable of deformation.^[^ ^78^ ^]^

In contrast, the intracellular route is not constrained by physical space but is influenced by the mechanisms of passive uptake. However, it is typically regarded as having minimum significance due to the corneocyte impermeability. In addition, the need for multiple partitioning and diffusion steps through the dense cell matrix makes this pathway even more challenging to penetrate. Once NPs reach the epidermis layer, pinocytosis, particularly clathrin‐mediated and caveolae‐mediated endocytosis, can occur. Smaller NPs, typically up to 100–150 nm, tend to be internalized through caveolae‐mediated endocytosis, while larger NPs, ranging from ≈200 to 5000 nm, are more likely to rely on clathrin‐mediated pathways.^[^ ^79^ ^]^ However, for pinocytosis to become a key mechanism for cellular uptake, NPs must first pass through the SC layer.

Size dependency arises not only from spatial constraints or endocytosis processes but also from NP aggregation. According to DLVO theory, though depending on the Hamaker constant (based on particle radius, surface speciation, temperature, and medium), larger NPs are generally more prone to aggregation due to increased *V*_vdW_^[^ ^70^ ^]^ despite their smaller surface area. Conversely, smaller NPs experience increased Brownian motion,^[^ ^80^ ^]^ which maintains dispersion but can also bring particles close to aggregate depending on the interplay of attractive and repulsive forces.^[^ ^81^ ^]^ Furthermore, their repulsive forces caused by hydration shells remain more effective compared to larger NPs due to higher curvature.^[^ ^82^ ^]^

The concentration of NPs also influences skin penetration across all layers, with the effect being slightly reduced for distant penetration. Some studies have demonstrated that higher concentrations and longer exposures enhance penetration.^[^ ^73^, ^83^ ^]^ However, at very high concentrations, NPs aggregation increases,^[^ ^70^ ^]^ reducing skin permeability. Additionally, the skin may become saturated with NPs beyond a certain threshold, limiting further absorption.

Surface‐to‐volume ratio showed relatively low significance, along with volume and surface area, which is notable because one of the key characteristics of NPs is their large surface‐to‐volume ratio, offering a significant interface with their surroundings. Our study highlights the importance of small NPs diameters, while other parameters related to NP dimensions, such as surface area and volume, were less impactful for skin penetration.

Medium showed the least importance among all the considered parameters describing NPs. While this parameter includes sub‐factors (e.g., pH, zeta potential, electrolyte valence), that affect the particle interaction energy, particularly the Hamaker constant and Debye–Hückel parameter, we were unable to include them due to insufficient data in the collected studies. Instead, we categorized the medium into broad qualitative classifications: aqueous, oil‐based, emulsion, or other types. This might be a reason for the low significance. Nonetheless, regarding the aggregation, media with a polar nature, such as water, which accounted for more than half of our dataset, facilitates the aggregation of small NPs.^[^ ^84^ ^]^ This accounts for increased surface energy, which the particles minimize by clustering together.^[^ ^81^ ^]^ This strong attractive *V*_vdW_ between particles coupled with random collisions arising from Brownian motions – particularly in small NPs – can lead to aggregation by shortening the distance between particles.^[^ ^81^ ^]^ Furthermore, differences in medium viscosity have an effect.^[^ ^85^ ^]^ A high‐viscosity medium, such as an emulsion, slows down the aggregation rate compared to a low‐viscosity medium like an aqueous solvent.

#### 3.2.3 Experimental Conditions Effect

Parameters of skin properties influencing NP diffusion through the skin are depicted in Figure 3B‐Experimental conditions.

Temperature was the most significant factor among all the experimental conditions considered. The temperature here refers to the entire experimental setup in the case of ex vivo studies, whereas in vivo temperature indicates the body temperature specifically. In this analysis, while the temperature of in vivo studies is body temperature, ex vivo assays cover temperatures between 22 and 37 °C. When considering the effect of temperature, both NPs and skin properties should be considered. For NPs, temperature affects the Hamaker constant, which in turn influences the magnitude of the *V*_vdW_ and the Debye–Hückel parameter. Increasing temperature results in a longer Debye–Hückel length, reducing *V*_EDL._^[^ ^86^ ^]^ The effect on the skin appears in the structure of the SC lipids, that is, as the temperature increases, the rigid, ordered structure of lipids in the SC becomes more disordered and flexible.^[^ ^87^ ^]^ This transition enhances the fluidity of the lipid matrix, making it easier for NPs to penetrate the skin. Additionally, as the temperature increases, the epidermis can lose moisture, leading to changes in skin barrier function,^[^ ^88^ ^]^ which could alter NP penetration pathways.

Ex vivo versus in vivo did not show significant effects, suggesting that whether NPs are tested on whole organisms or isolated skin samples, the outcomes remain broadly consistent, with only minimal influence on the results. But instead, the temperature of the experimental condition in the case of ex vivo should be carefully chosen.

Contact time has already been a topic of ongoing discussion.^[^ ^89^, ^90^ ^]^ Our dataset included a range of contact times, from 15 min to one week, which suggests that prolonged NPs contact does not significantly improve penetration (Figure 4F).

#### 3.2.4 Skin Properties Effect

Figure 3B Skin illustrates the skin properties influencing transdermal transport, with hair follicle diameter emerging as the most influential parameter. This parameter becomes particularly crucial when targeting NPs to penetrate below the epidermis, as transappendegeal route penetration is typically dermal. As depicted in Figure 4A, hair follicle diameters significantly vary across species and body regions, ranging from 14 µm in rat back to 185 µm in pig abdomen. In humans, diameters are relatively consistent across regions, with similar sizes in the face (71.3 µm), abdomen (97 µm) arm (103.2 µm), back (140.3 µm), and breast (146.3 µm). Compared to the other species, the hair follicle diameter in the human abdomen and arm, often used in in vivo experiments, most closely match those of rat abdomen and pig ear. The human breast, commonly used in ex vivo NP skin penetration studies, has a hair follicle diameter that most closely resembles a pig ear. These findings highlight the importance of selecting the appropriate specimen based on species and body region. While skin thickness has traditionally been emphasized, with pig skin being a common alternative,^[^ ^25^, ^91^, ^92^ ^]^ hair follicle diameter should be recognized as an even more critical factor for NPs skin penetration study. In contrast, hair follicle density appears to have a limited impact with the importance of variables <5 (Figure 3B Skin), suggesting that NPs penetration is not strongly correlated with hair follicle quantity.

Thickness of SC, epidermis, and dermis are essential factors influencing NPs diffusion. For NPs to penetrate, both the thickness of each layer and the one above it are essential factors according to our results. Thicker layers require NPs to travel a greater distance, making penetration more challenging. Although the SC is only ≈10–20 µm thick, the intercellular diffusion path is ≈20 times longer due to its tortuous structure.^[^ ^77^ ^]^

While the lipophilicity of SC lipids showed relatively low importance with <10 (Figure 3B Skin), it remains an interesting parameter to consider, particularly in terms of retaining NPs on the surface or facilitating their delivery into the layers beneath the SC. The intercellular route primarily supports the transport of hydrophobic or non‐polar solutes through the lipid‐rich spaces between skin cells. Thus, changes in SC lipid lipophilicity could affect the efficiency of this transport route. The implications of these changes on the intercellular transport pathway are discussed further in the next section. On the other hand, the polarity of SC lipids has minimal significance without layer‐specific variables.

Species parameters showed a low substantial effect, indicating that the choice of species for NP skin penetration studies is less important than selecting the appropriate body region. Key factors such as hair follicle diameter, skin thickness, and the lipophilicity of the SC lipids should be considered to match the skin characteristics with the specific goals for delivering NPs effectively.

### 3.3 Influence of Species and Skin‐Region

Given the importance of the skin in the dermal delivery of NPs, it is evident that NP penetration results vary significantly depending on the species and the specific skin region studied. With only ≈24% of studies using human skin (Figure 2D, Skin), a question arises: how can we compare NP penetration across different species and body regions to yield results relevant for human use? To address this, we analyzed factors such as anatomical differences across species and body regions in relation to NP transdermal penetration. In Figure 4, we present the influence of a spectrum of NP parameters on the penetration depth, sorted by skin layer and skin region of humans.

Figure 4B illustrates the skin thickness of the back region, including the SC, epidermis, dermis, hair follicle diameter from mice, rats, pigs, and humans. The SC thickness of humans is closest to that of the mouse (+1.7%), followed by rabbit (+19%), pig (+56.9%), and rat (+110.8%). Thus, we can expect slight difficulties in penetrating the outermost layer SC for the commonly used pig skin compared to human skin. In the case of the rat, this is especially significant as its SC is more than twice as thick as that of humans. Human skin is the thickest of all species for the epidermis and dermis. This may indicate that once NPs penetrate the SC, easier delivery in pigs, rabbits, mice, and rats could be expected compared to humans. The human epidermis thickness is closest to pig, followed by rabbit, rat, and mouse (−29.1%, −43.2%, −65.5%, and −77.8%, respectively). Among all skin layers, the dermis thickness of animal species considered shows the least similarity to humans, with rabbit (−49.5%) being the closest, followed by pig (−59.3%), rat (−84.8%), and mouse (−95.9%). In terms of hair follicle diameter, human has the largest diameter compared to pigs, mice, and rats, with significant margins of −73%, −82.5%, and −90%, respectively, which may suggest more efficient transappendageal route delivery in human skin when used for NP penetration assays. Figure 4C illustrates the overall similarity in skin thickness in the back region across species. Pig skin is the closest to human skin, followed closely by rabbits. This aligns with the common use of pig skin as a substitute for human skin owing to similarities in thickness, hair follicle structure, and sweat glands.^[^ ^92^ ^]^ However, our analysis does not include other parameters apart from skin thickness (e.g., hair follicle diameter or density) due to data limitations though hair follicle density especially shows small significance (Figure 3B Skin). Detailed results for similarity in each skin layer are provided in Figure S5 (Supporting Information).

Having understood that the degree of penetration of NPs varies significantly based on their size and hydrophobicity, as well as the specific skin region being analyzed, we have investigated the relationship between NP size and hydrophobicity across different skin areas. Figure 4D illustrates the size distribution of NPs in various skin layers, that are often penetrated. Interestingly, different layers exhibit preferences for distinct NP sizes within a single skin region. For example, deeper layers require smaller particles for effective penetration, particularly in areas like the abdomen. However, other skin regions do not consistently follow this pattern; in some cases, deeper layers allow larger NPs to penetrate than shallower ones. This suggests that once NPs penetrate the SC, larger particles may be more likely to continue their journey into the skin. Small particles travel through more extended and more complex diffusion pathways in the tortuous structure of SC, compared to larger particles, which is analogous to the principle of size exclusion chromatography.

While the size of NPs showed clear independent significance, the hydrophobicity distribution (Figure 4E) did not reveal a clear preference for specific hydrophobicity levels in penetrating particular skin layers. This indicates that a wide range of hydrophobicity can potentially facilitate the delivery of NPs into the skin. However, our previous analysis demonstrated that hydrophobicity is one of the most critical parameters for dermal delivery of NPs (Figure 3B, NPs). Thus, hydrophobicity likely becomes a particularly significant factor when considered in conjunction with other variables, such as NP size, concentration, the type of medium used, and temperature as opposed to NP size exhibiting an independent significance.

### 3.4 What Route Do NPs Take?

Understanding the routes through which NPs penetrate the skin would notably support developing targeted delivery systems. Bearing that in mind, we used the in‐silico human model to determine the penetration behavior of NPs by comparing different skin parameters under the following conditions: 0) Normal skin (baseline, no changes), and 1) reduced number of hair follicle routes, 2) increased lipophilicity and decreased polarity of the SC lipid or 3) decreased lipophilicity and increased polarity of the SC lipid. These parameters were altered incrementally by 5%, 10%, 15%, and 20% to examine the gradient effects on the efficiency of NP penetration. For the purpose of the analysis, 100 000 generated NPs were split into three groups defined by commonly used media: aqueous solution, emulsion, and oil.

Scenario (1) (reduced hair follicle routes) allowed us to observe how the reduced number of hair follicles impacts NP penetration, especially in identifying the significance of transappendageal routes for NP embedded in different media. Scenario (2) (increased lipophilicity and decreased polarity of SC lipids) was expected to enhance the intercellular route, facilitating the passage of NPs by making the lipid matrix more favorable for their transport. Scenario (3) (decreased lipophilicity and increased polarity of SC lipids) was hypothesized to shift the emphasis toward intracellular pathways, as the altered lipid environment would inhibit NPs from traversing the intercellular route. By comparing the NP penetration behavior under these scenarios (1–3) to the baseline (0), we aim to reveal the varied pathways and mechanisms that influence NP transport through the skin layers. It should be noted that these shifts indicate the preferred pathways and do not indicate that particular routes are exclusively used.

**Figure 5:**
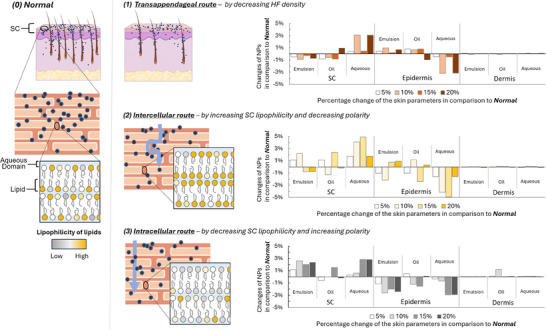
The analysis of NPs penetration routes using in‐silico humans and NPs. The comparison of the baseline scenario (0) with scenarios (1)–(3) reveals the emphasized pathways and mechanisms influencing NP transport through skin layers. Scenario (1) examines the effect of reduced hair follicle density, emphasizing the role of transappendageal routes across different media. Scenario (2) explores the impact of increased SC lipid lipophilicity, which is expected to enhance the intercellular route and facilitate transport. Scenario (3) investigates decreased SC lipid lipophilicity, potentially shifting transport toward intracellular pathways by limiting intercellular movement. The figure on the right illustrates the percentage of NPs penetrating the SC, epidermis, and dermis under each scenario in comparison to the baseline (0). Elements are drawn with the assistance of BioRender.

The results of the analysis are presented in **Figure** **5**. Across all penetration routes, the penetration of SC and epidermis showed the greatest sensitivity to changes in NP parameters, while the dermis was minimally affected, with changes of less than 1% compared to the baseline (0 – no changes to skin parameters). Considering the transappendageal route, after a reduction in hair follicle density NPs embedded in aqueous media persisted within the SC, while those embedded in emulsion or oil exhibited reduced accumulation. Further, NPs from aqueous media struggled to reach the epidermis, while those from an emulsion or oil media overcame the SC barrier and accumulated in the epidermis. A similar trend was observed in the intercellular route, with aqueous media showing reduced efficiency compared to penetration of NPs from emulsion or oil. The effect of aqueous media is more pronounced for the intercellular route than transappendageal, indicating that the former is less favorable for aqueous formulations. The significant influence of skin parameter changes on the penetration from all three media underscores the importance of the intercellular path as a primary route for NP transport. In the intracellular route, emulsion and aqueous media were particularly affected, showing increased accumulation in the SC and reduced penetration into the epidermis compared to the baseline. This result suggests that the intracellular pathway is inherently challenging for NPs regardless of the medium.

In summary, NPs embedded in aqueous media demonstrated slightly greater ease in penetrating via the intracellular route but were generally the least affected by medium type, while those embedded in emulsion or oil media predominantly utilized the transappendageal and intercellular pathways. This is likely because the intercellular pathway requires NPs to diffuse through the lipid matrix surrounding the corneocytes in the SC to reach deeper layers of the skin, a process more compatible with lipophilic media like emulsions and oils. Similarly, the transappendageal route, characterized by tightly packed keratinized corneocytes at the surface of the follicular opening,^[^ ^93^ ^]^ may facilitate the penetration of emulsion or oil‐based NPs deeper into the skin.

### 3.5 Limitations of the Model

The data on NPs, skin, experimental conditions, and efficiency of NP penetration are taken from diverse publications. Many studies on NP penetration through skin provided partial information, typically including details only on the NPs (material, size, medium –, e.g., water, emulsion), skin type (species), and testing conditions (e.g., in vivo vs in vitro). As a result, a substantial amount of supplementary data was incorporated from other sources based on the available information, for example, data on skin layer thickness and NP hydrophobicity. This supplementation introduces a potential deviation between the experimental data and the calculated results due to additional parameters used in our analysis. Furthermore, specific parameters, such as pH or zeta potential of the medium, were excluded due to scarce reporting in the literature.

Although we successfully demonstrated the potential routes of NP skin penetration from different media, the analysis of intracellular routes could have been enhanced by additional data on keratinocytes in the epidermis across species if only available in literature in a sufficient number.

## 4 Conclusion

We have presented for the first time a comprehensive in‐silico analysis of NP penetration through skin, considering NP characteristics, skin types, properties, and experimental conditions, based on a two‐decade compilation of data from studies across various species.

Our findings resulted in three key outcomes. First, we successfully developed a predictive model to analyze a complex inter‐species model of NP skin penetration. The Random Forest algorithm and the Kennard‐Stone sorting method achieved the highest predictive accuracy. Thereafter, we highlighted the most influential factors affecting NP penetration through the skin, with hair follicle diameter emerging as the most dominant variable for penetration across all skin layers. This factor was more significant than any NP characteristic or experimental condition, with NP hydrophobicity and core diameter being the most influential variables. This result highlights the importance of selecting an appropriate skin model for specific experimental goals. In NP penetration studies, pig and rabbit skin are the best alternatives for simulating human skin. Last, we identified routes of NP penetration using the developed in‐silico human model. The results showed that NPs in emulsions or oil‐based media primarily penetrate via the intercellular and transappendageal routes.

In contrast, NPs in aqueous media were slightly more efficient at penetrating the intracellular route. However, penetration of this route was less affected by medium variations. These findings offer valuable insights for designing NPs targeted to penetrate specific skin layers or prevent unintended skin penetration by NPs.

## Conflict of Interest

The authors declare no conflict of interest.

## Author Contributions

N.M. dealt with conceptualization, methodology, software, validation, formal analysis, investigation, data curation, writing the original draft, writing the review and editing, and visualization. H.J. dealt with methodology, validation, investigation, data curation, and visualization. I.E.K.‐C. dealt with conceptualization, investigation, writing the original draft, and writing the review and editing. W.A. dealt with methodology, software, validation, writing the original draft, and writing the review and editing. U.P. dealt with writing the original draft, and writing the review and editing. P.S. dealt with writing the original draft, writing the review and editing. A.M. dealt with writing the original draft, writing the review and editing. C.J. dealt with conceptualization, methodology, software, validation, formal analysis, data curation, writing the original draft, writing the review and editing, supervision, and project administration.

## Supporting information



Supporting Information

Supplemental Table 1

Supplemental Table 2

## Data Availability

The data that support the findings of this study are available in the supplementary material of this article.
