# Supplementary material for: Nanoparticle Skin Penetration: Depths and Routes Modeled In‐Silico
Source: Small. 2025 Mar 27;21(20):2412541. doi: 10.1002/smll.202412541 (PMC12087856; doi:10.1002/smll.202412541)
Supplement: Supplementary file 1 — Supporting Information [file SMLL-21-2412541-s001.docx]

**Supporting Information (SI)**

Nanoparticle Skin Penetration: Depths and Routes Modeled In-Silico

Natsumi Maeda^a^, Haixin Jiao^b^, Ilona Edyta Kłosowska-Chomiczewska^c^, Wojciech Artichowicz^d^, Ulrich Preiss^e^, Patrycja Szumała^c^, Adam Macierzanka^c^, and Christian Jungnickel^c*^

^a^ Institute of Biogeochemistry and Pollutant Dynamics, Swiss Federal Institute of Technology, ETH Zürich, Universitätstrasse 16, 8092 Zürich, Switzerland

^b^ Biofuels Institute, School of the Environment and Safety Engineering, Jiangsu University, Zhenjiang 212013, Peoples Republic of China

^c^ Department of Biotechnology and Microbiology, Faculty of Chemistry, Gdańsk University of Technology, Narutowicza 11/12, 80-233 Gdańsk, Poland

^d^ Department of Hydraulic Engineering, Faculty of Civil and Environmental Engineering, Gdańsk University of Technology, Narutowicza 11/12, 80-233, Gdańsk, Poland

^e^ Omya GmbH, Siegburger Str. 229c, 50679 Köln, Germany

*Corresponding author: [christian.jungnickel@pg.edu.pl](mailto:christian.jungnickel@pg.edu.pl), tel: +48 58 347 2469


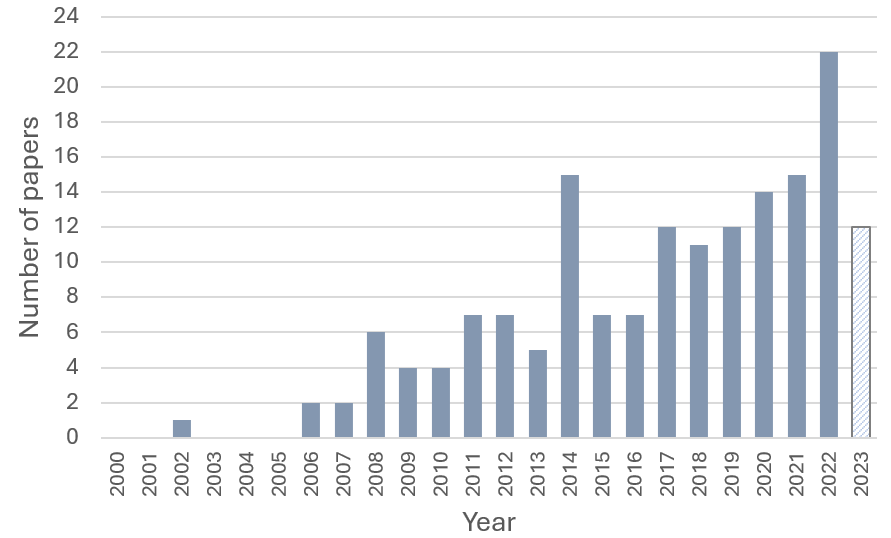


**Figure S1.** The papers collected from Google Scholar using consistent search terms of *"skin penetration" AND "nanoparticles" AND "stratum corneum" AND "histology" -microneedle -reviews -NLC -SLN -liposome -DNA -RNA "vivo" OR "vitro"*. It demonstrates that research on nanoparticle skin penetration has been increasing over the past 20 years. The data for 2023 is incomplete, as the papers were collected during that year.

*Table S1 and S2 are attached as separate files*.

**Table S1** contains all the NP data used in the model, including experimental conditions of the skin penetration tests

**Table S2** contains the skin data from the species which was used to generate the average skin data for each species. Included is also the body part from which the skin was sampled.


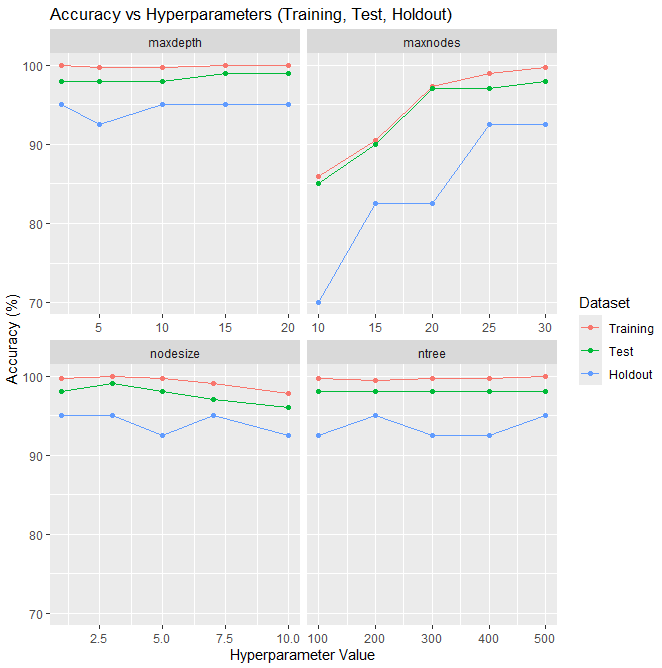


**Figure S2.** Hyperparameter optimization results for the random forest calculations. The hyperparameters were calculated using a grid search type approach, with 625 possible variations. The accuracy of each parameter for training, testing, and holdout is shown. Reduced accuracy in the holdout data, while training/test accuracy was high, indicated overfitting. The chosen hyperparameters were *maxdepth* 20, *maxnode* 25, *nodesize* 1, and *ntree* 500.


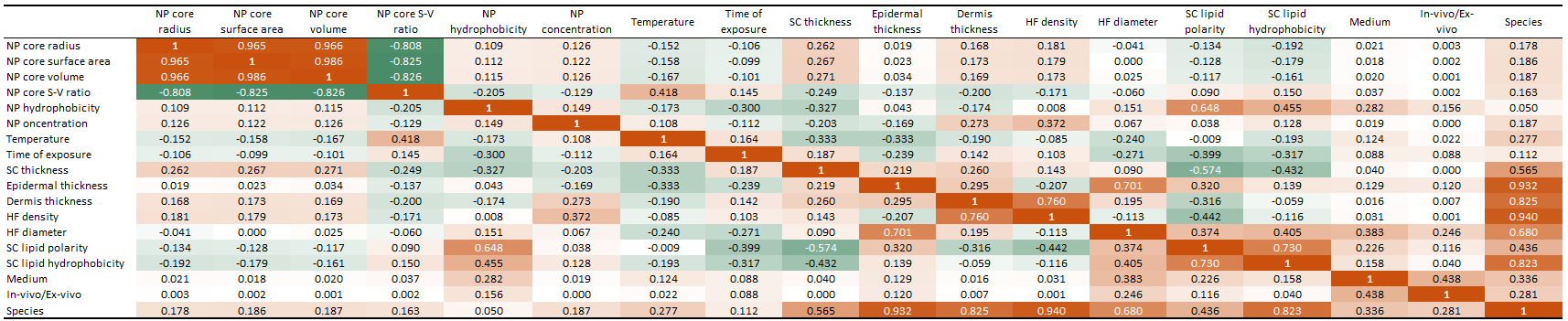


**Table S3.** Correlation matrix of the complete skin data. Switches are not shown.


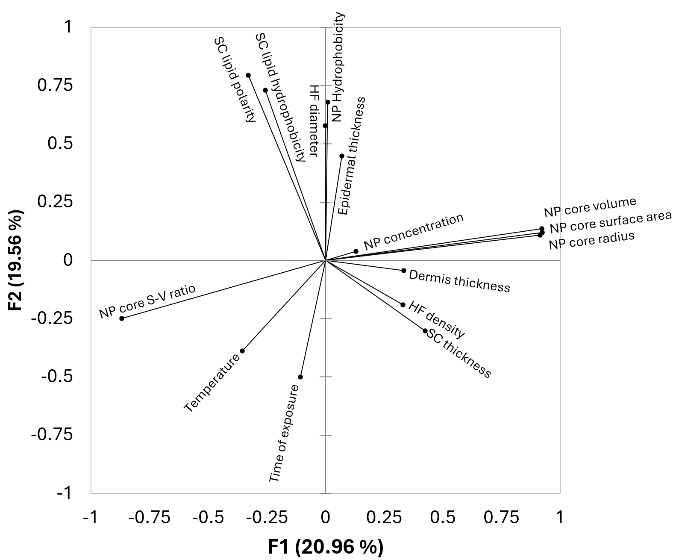


**Figure S3.** Principal Component Analysis (PCA) of the quantitative data. Similar contributions to the model of the SC lipid hydrophobicity and lipid polarity are evident, and all the NP geometric data overlap. Interestingly, HF diameter and NP hydrophobicity showed a substantial overlap.


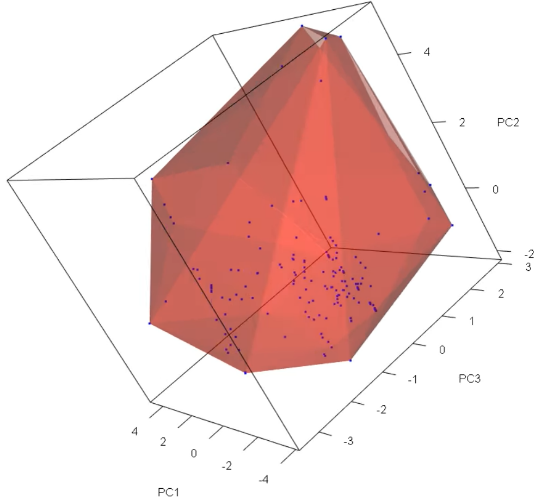


**Figure S4.** Convex hull representation of the applicability domain of the dataset used to create the model.


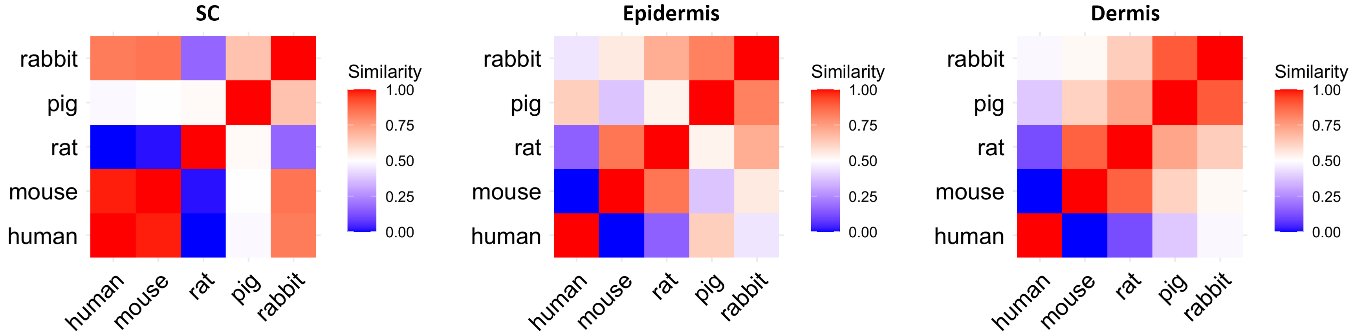


**Figure S5.** The heatmap depicts the similarity in skin thickness across different species in the back region, at the SC, epidermis, and dermis layers. The Euclidean distance between values was calculated using the dist function in R.
